# Hospital admission after primary care consultation for community-onset lower urinary tract infection: a cohort study of risks and predictors using linked data

**DOI:** 10.3399/BJGP.2022.0592

**Published:** 2023-07-25

**Authors:** Anna Aryee, Patrick Rockenschaub, John Robson, Marian Priebe, Zaheer Ahmed, Caoimhe Nic Fhogartaigh, David Ball, Andrew Hayward, Laura Shallcross

**Affiliations:** Institute of Health Informatics, University College London, London.; Institute of Health Informatics, University College London, London.; Clinical Effectiveness Group, Wolfson Institute of Population Health, Queen Mary University of London, London.; Clinical Effectiveness Group, Wolfson Institute of Population Health, Queen Mary University of London, London.; Clinical Effectiveness Group, Wolfson Institute of Population Health, Queen Mary University of London, London.; Barts Health NHS Trust, London.; Barts Health NHS Trust, London.; Institute of Epidemiology and Health Care, University College London, London.; Institute of Health Informatics, University College London, London.

**Keywords:** antimicrobial stewardship, cohort studies, primary care, secondary care, urinary tract infections

## Abstract

**Background:**

Urinary tract infections (UTIs) are a common indication for antibiotic prescriptions, reductions in which would reduce antimicrobial resistance (AMR). Risk stratification of patients allows reductions to be made safely.

**Aim:**

To identify risk factors for hospital admission following UTI, to inform targeted antibiotic stewardship.

**Design and setting:**

Retrospective cohort study of East London primary care patients.

**Method:**

Hospital admission outcomes following primary care consultation for UTI were analysed using linked data from primary care, secondary care, and microbiology, from 1 April 2012 to 31 March 2017. The outcomes analysed were urinary infection-related hospital admission (UHA) and all-cause hospital admission (AHA) within 30 days of UTI in primary care. Odds ratios between specific variables (demographic characteristics, prior antibiotic exposure, and comorbidities) and the outcomes were predicted using generalised estimating equations, and fitted to a final multivariable model including all variables with a *P*-value <0.1 on univariable analysis.

**Results:**

Of the 169 524 episodes of UTI, UHA occurred in 1336 cases (0.8%, 95% confidence interval [CI] = 0.7 to 0.8) and AHA in 6516 cases (3.8%, 95% CI = 3.8 to 3.9). On multivariable analysis, increased odds of UHA were seen in patients aged 55–74 years (adjusted odds ratio [AOR] 1.49) and ≥75 years (AOR 3.24), relative to adults aged 16–34 years. Increased odds of UHA were also associated with chronic kidney disease (CKD; AOR 1.55), urinary catheters (AOR 2.01), prior antibiotics (AOR 1.38 for ≥3 courses), recurrent UTI (AOR 1.33), faecal incontinence (FI; AOR 1.47), and diabetes mellitus (DM; AOR 1.37).

**Conclusion:**

Urinary infection-related hospital admission after primary care consultation for community-onset lower UTI was rare; however, increased odds for UHA were observed for some patient groups. Efforts to reduce antibiotic prescribing for suspected UTI should focus on patients aged <55 years without risk factors for complicated UTI, recurrent UTI, DM, or FI.

## INTRODUCTION

Urinary tract infections (UTIs) are one of the commonest indications for antibiotic prescriptions in primary and secondary care, and reduction in prescribing for UTI would likely have a significant impact on overall antibiotic consumption and rates of antimicrobial resistance (AMR). Estimates suggest that the average person consults their GP 5.5 times per year and that 1%–3% of all GP consultations are for UTI symptoms.^1^^,^^2^ In addition to symptomatic relief, fear of complications (for example, pyelonephritis [upper UTI], urinary sepsis, and bloodstream infection) is an important driver in antibiotic prescribing for lower UTI.^3^^–^5

Studies suggest that a proportion of uncomplicated UTIs are self-limiting, with up to 50% of women being symptom free without antibiotic treatment at 7 days.^6^^,^^7^ Antibiotic use is also associated with the development of AMR, gastrointestinal side effects, and complications such as *Clostridium difficile* colitis.^8^^,^^9^ Delay or avoidance of antibiotic treatment may therefore be preferable for certain patients. Trials involving young women have found that approximately two-thirds of patients recover with symptomatic treatment rather than antibiotics, but that this strategy was associated with a higher symptom burden and, in one of the studies, more cases of pyelonephritis.^10^^,^^11^ Such approaches may reduce antibiotic consumption, but data on patient outcomes are necessary to inform their acceptability and safety. A recent study using electronic health records (EHRs) found that the probability of sepsis was higher following consultations for UTI than for respiratory tract or skin infections, and that the risk of sepsis was higher among older adults.^12^ Other analyses of large datasets using EHRs have found conflicting results in older adults.^13^^,^^14^

The aim of this study was to obtain an accurate estimate of the risk of adverse outcomes following lower UTI in primary care in patients aged ≥16 years, in order to identify patients for whom antibiotic treatment could be safely delayed or avoided as a means of antibiotic stewardship.

## METHOD

This was a retrospective cohort study using primary care EHRs linked to secondary care and microbiology data.

### Data source

The study cohort was created using a primary care database of East London general practices developed and managed by the Clinical Effectiveness Group (CEG), part of Queen Mary University of London. The data were deterministically linked to Secondary Uses Services (SUS, managed by NHS Digital) secondary care data, and to microbiology data (urine and blood cultures) from Barts Health NHS Trust as per the supplementary material.

**Table:** How this fits in

Reductions in antibiotic prescribing for urinary tract infections (UTIs) would reduce overall antibiotic consumption and antimicrobial resistance. Previous studies on prescribing using routinely collected data have previously not included microbiology data. Linkage of microbiology data to primary care and secondary care data is a novel element of this study that allowed the authors to strengthen the outcomes of urinary infection-related hospital admission in a way that has not been previously described. This study found that antibiotic stewardship efforts for UTIs should be targeted at younger patients without specific risk factors.

Consultations for lower UTI were identified through Read codes, antibiotic prescriptions for UTI, and positive urine cultures with relevant uropathogens. This study employed a modified version of the Read code lists used by previous similar studies (see Supplementary Table S2).^13^^,^^14^ First-line antibiotics used to treat lower UTI are frequently unlinked to a diagnostic code, so UTI consultations were also identified through prescriptions for nitrofurantoin, trimethoprim, fosfomycin, and pivmecillinam.^15^ Relevant uropathogens are shown in Supplementary Table S3. UK guidelines recommend sending urine cultures only in certain situations including complicated UTI, treatment failure, or where antibiotic resistance is suspected.^16^ To ensure that the data set did not include patients with upper UTI, consultations where a Read code for upper UTI was recorded within +/–3 days of a positive urine culture were excluded (see Supplementary Table S4). Distinct episodes of UTI were identified using a 30-day washout period, with any consultations within that period considered part of the same episode. Any consultations outside the washout period were considered a new episode. Any consultation occurring within the washout period was considered an ongoing episode and excluded from the analysis (see Supplementary Figure S1). Patients could have >1 UTI episode during the study period.

### Population

Patients aged ≥16 years registered at the approximately 100 GP surgeries across Tower Hamlets and Newham, London, who consulted their GP for UTI between 1 April 2012 and 31 March 2017 were eligible for inclusion in the study. Patients were excluded from the study if:

- there were no data available for sex, age, Index of Multiple Deprivation (IMD) score;
- they were registered for <12 months prior to their first episode (to allow for identification of comorbidities);
- there were <30 days’ follow-up data available (unless death occurred within that period);
- they were admitted to hospital on the day of their UTI episode; and
- they were discharged from hospital in the 30 days prior to their UTI episode.

Patients who had multiple episodes of UTI were included and entered the cohort at the start of their first episode. Patients left the cohort at the earliest of these dates: death, change of practice, or end of the study period.

### Putative risk factors

A number of risk factors (see supplementary material for rationale and definitions) were examined as variables in the analysis, including:

- demographics: age, sex, ethnicity, IMD quintile (obtained by linking individual patient’s postcode to Lower Layer Super Output Area);
- non-treatment with antibiotics within +/‒7 days;
- risk factors for complicated UTI: structural abnormalities, chronic kidney disease (CKD), urinary catheterisation;
- recurrent UTI;
- comorbidities: cancer, diabetes mellitus (DM), heart failure, hypertension, urinary incontinence (UI), faecal incontinence (FI), and obesity;
- antibiotic prescriptions in the last 6 months (see supplementary material); and
- season of the year.

### Outcomes

The primary outcome was urinary infection-related hospital admission (UHA) in the 30 days from the start of an episode. This definition included International Classification of Diseases, 10th Revision (ICD-10) codes related to UTI (upper UTI, sepsis, and bloodstream infection; Supplementary Table S9), and urine and blood cultures positive for relevant organisms within 2 days of hospital admission. The secondary outcome was all-cause hospital admission (AHA) in the 30 days following an episode.

### Statistical analyses

Descriptive statistics were used to summarise the clinical and demographic characteristics. Continuous variables were summarised using median and interquartile range (IQR), and categorical variables using absolute numbers and proportions. Crude associations (odds ratios [ORs]) were estimated between each of the variables (risk factors) and the outcomes using generalised estimating equations (GEEs) with a logit link and an exchangeable correlation structure to account for multiple episodes per patient. Huber‒White sandwich estimators were used to calculate 95% confidence intervals (95% CIs). A final multivariable adjusted model was fitted using GEEs, including all predictors with a *P-*value <0.1 in the univariable analysis. Age was included as a categorical variable in the multivariable model, as this was felt to be most informative for primary care prescribing decisions.

Older patients may be more likely to be treated with antibiotics because of concerns around progression to sepsis,^17^ so the possibility of an interaction between antibiotic treatment within +/–7 days and age on the outcome of UHA was explored. A model was run including an interaction term between age and antibiotic treatment, looking for a significant Wald *P*-value for the interaction coefficients. The Quasi Information Criterion (uQIC) was used to assess the model fit, using a difference in uQIC of ≥10 to signify a statistically significant improvement (where a lower number indicates a better fit). All data cleaning and analyses were performed using the statistical software R (version 3.6.1) for Windows. Generalised estimating equations were fitted using *geepack* (version 1.2-1).

## RESULTS

**Figure 1.**
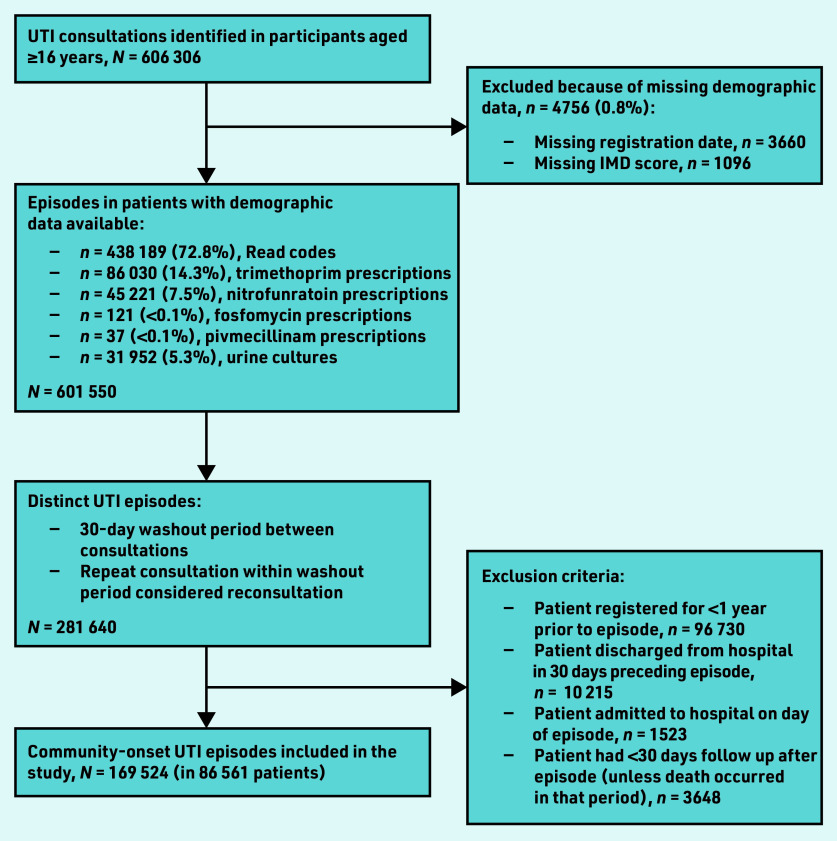
*Flowchart of creation of cohort. IMD = Index of Multiple Deprivation. UTI = urinary tract infection.*

A total of 169 524 UTI episodes (in 86 561 patients) were included in the study (Figure 1). The majority of episodes (*n* = 132 094; 77.9%) occurred in female patients, and the median age was 43 years (IQR 31–60; see Supplementary Table S1). Factors associated with antibiotic treatment within +/–7 days of the episode were female sex, older age, increased socioeconomic deprivation, prior antibiotic treatment, risk factors for complicated UTI, and all other comorbidities examined.

**Figure 2.**
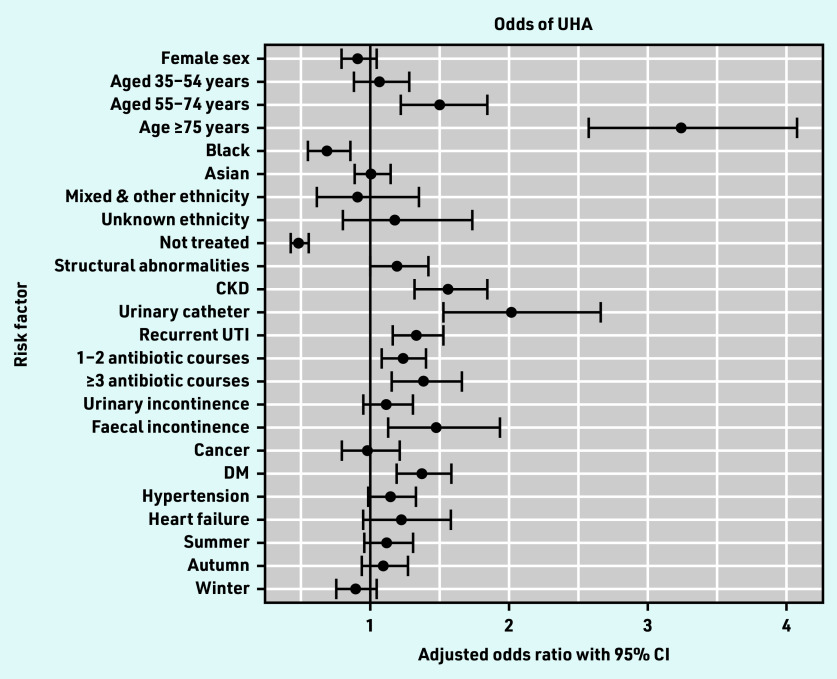
*Forest plot of multivariable analysis of odds of UHA. CKD = chronic kidney disease. DM = diabetes mellitus. UHA = urinary infection-related hospital admission. UTI = urinary tract infection.*

Of the 169 524 UTI episodes, UHA occurred in 1336 episodes (0.8%, 95% CI = 0.7 to 0.8). On multivariable analysis adjusting for age, sex, antibiotic treatment, and all variables associated with the outcome on univariable analysis, the factor most strongly associated with UHA was older age (Figure 2). Patients in the age groups 55–74 years and ≥75 years had increased odds of UHA as compared with those aged 16–34 years, with adjusted odds ratios (AORs) of 1.49 (95% CI = 1.21 to 1.84) and 3.24 (95% CI = 2.57 to 4.08), respectively (Table 1).

**Table 1.** Multivariable analysis of odds of UHA

**Characteristic**	**Adjusted odds ratio (95% CI)**	***P*-value**
**Sex**		
Male	1	
Female	0.91 (0.79 to 1.04)	0.167

**Age (categorical), years**		
16–34	1	
35–54	1.06 (0.88 to 1.28)	0.545
55–74	1.49 (1.21 to 1.84)	<0.001
≥75	3.24 (2.57 to 4.08)	<0.001

**Ethnicity**		
White	1	
Black	0.68 (0.55 to 0.85)	<0.001
Asian	1.0 (0.88 to 1.14)	0.946
Mixed and other	0.91 (0.61 to 1.35)	0.627
Unknown	1.17 (0.80 to 1.73)	0.417

**Treatment within +/−7 days**		
Treated	1	
Not treated	0.48 (0.42 to 0.55)	<0.001

**Antibiotics last 6 months, courses**		
0	1	
1–2	1.23 (1.08 to 1.40)	0.002
≥3	1.38 (1.15 to 1.65)	<0.001

**Risk factors for complicated UTI**		
Absence of risk factor	1	
Structural abnormalities	1.19 (1.00 to 1.41)	0.051
CKD	1.55 (1.31 to 1.84)	<0.001
Urinary catheter	2.01 (1.53 to 2.66)	<0.001

**Other risk factors**		
Absence of risk factor	1	
Recurrent UTI	1.33 (1.16 to 1.53)	<0.001
UI	1.11 (0.94 to 1.30)	0.208
FI	1.47 (1.12 to 1.93)	0.005
Heart failure	1.22 (0.95 to 1.58)	0.125
Hypertension	1.14 (0.98 to 1.33)	0.091
Cancer	0.98 (0.79 to 1.21)	0.825
DM	1.37 (1.19 to 1.58)	<0.001

**Season**		
Spring	1	
Summer	1.11 (0.95 to 1.30)	0.173
Autumn	1.09 (0.93 to 1.27)	0.273
Winter	0.89 (0.75 to 1.04)	0.144

*CKD = chronic kidney disease. DM = diabetes mellitus. FI = faecal incontinence. IMD = Index of Multiple Deprivation. UHA = urinary infection-related hospital admission. UI = urinary incontinence. UTI = urinary tract infection.*

Recurrent UTI (AOR 1.33; 95% CI = 1.16 to 1.53), CKD (AOR 1.55; 95% CI = 1.31 to 1.84), and urinary catheters (AOR 2.01; 95% CI = 1.53 to 2.66) were associated with increased odds of UHA (Table 1). Of other comorbidities examined, only FI and DM were associated with increased odds of UHA, with AORs of 1.47 (95% CI = 1.12 to 1.93) and 1.37 (95% CI = 1.19 to 1.58), respectively. Prior antibiotics were also associated with increased odds of UHA, with AORs of 1.23 (95% CI = 1.08 to 1.40) for 1–2 courses and 1.38 (95% CI = 1.15 to 1.65) for ≥3 courses, compared with no prior antibiotic courses.

**Figure 3.**
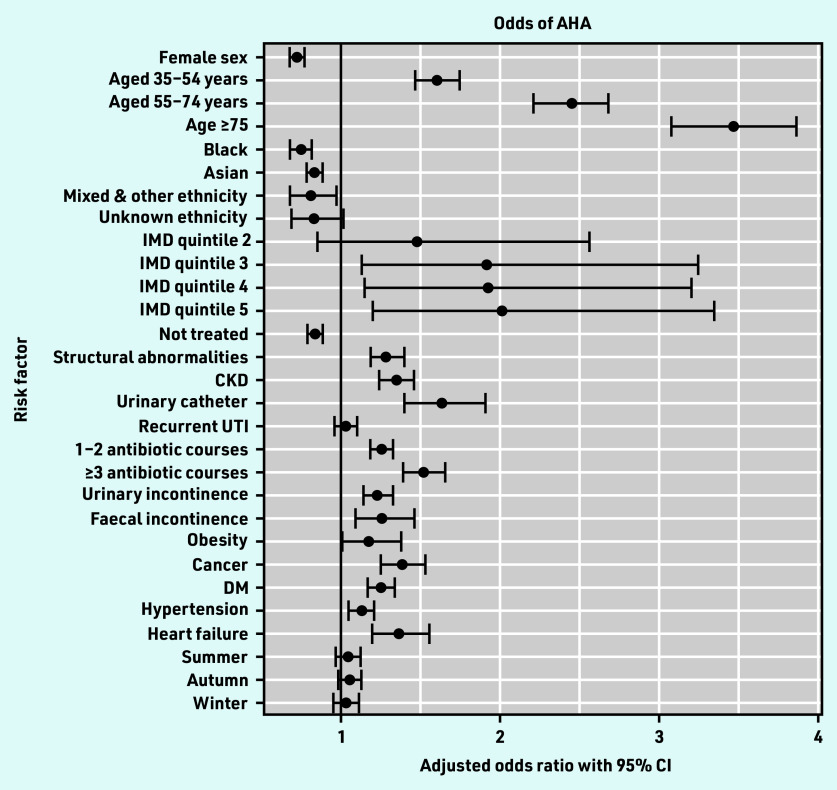
*Forest plot of multivariable analysis of odds of AHA. AHA = all-cause hospital admission. CKD = chronic kidney disease. DM = diabetes mellitus. IMD = Index of Multiple Deprivation. UTI = urinary tract infection.*

Of the 169 524 UTI episodes, AHA occurred in 6516 episodes (3.8%, 95% CI = 3.8 to 3.9). On multivariable analysis adjusting for age, sex, antibiotic treatment, and all variables associated with the outcome on multivariable analysis, factors associated with increased odds of AHA included older age, increased socioeconomic deprivation, prior antibiotic exposure, risk factors for complicated UTI, and all comorbidities examined apart from recurrent UTI (Table 2 and Figure 3).

**Table 2.** Multivariable analysis of odds of AHA

**Characteristic**	**Adjusted odds ratio (95% CI)**	***P*-value**
**Sex**		
Male	1	
Female	0.73 (0.68 to 0.77)	<0.001

**Age (categorical), years**		
16–34	1	
35–54	1.61 (1.47 to 1.75)	<0.001
55–74	2.45 (2.22 to 2.70)	<0.001
≥75	3.47 (3.09 to 3.89)	<0.001

**IMD quintile**		
1 (least deprived)	1	
2	1.48 (0.86 to 2.58)	0.16
3	1.93 (1.14 to 3.26)	0.015
4	1.93 (1.15 to 3.22)	0.012
5 (most deprived)	2.02 (1.21 to 3.37)	0.007

**Ethnicity**		
White	1	
Black	0.75 (0.68 to 0.82)	<0.001
Asian	0.83 (0.78 to 0.89)	<0.001
Mixed & other	0.81 (0.68 to 0.97)	0.025
Unknown	0.84 (0.69 to 1.02)	0.079

**Treatment within +/−7 days**		
Treated	1	
Not treated	0.84 (0.79 to 0.89)	<0.001

**Risk factors for complicated UTI**		
Absence of risk factor	1	
Structural abnormalities	1.29 (1.19 to 1.41)	<0.001
CKD	1.35 (1.24 to 1.47)	<0.001
Urinary catheter	1.64 (1.40 to 1.91)	<0.001

**Antibiotics last 6 months, courses**		
0	1	
1–2	1.26 (1.19 to 1.34)	<0.001
≥3	1.52 (1.40 to 1.66)	<0.001

**Other risk factors**		
Absence of risk factor	1	
Recurrent UTI	1.03 (0.96 to 1.10)	0.404
UI	1.24 (1.15 to 1.33)	<0.001
FI	1.27 (1.09 to 1.47)	0.002
Obesity	1.19 (1.02 to 1.39)	0.025
Heart failure	1.37 (1.21 to 1.56)	<0.001
Hypertension	1.13 (1.06 to 1.21)	<0.001
Cancer	1.39 (1.26 to 1.54)	<0.001
DM	1.26 (1.17 to 1.34)	<0.001

**Season**		
Spring	1	
Summer	1.04 (0.97 to 1.12)	0.239
Autumn	1.05 (0.98 to 1.13)	0.144
Winter	1.03 (0.96 to 1.11)	0.421

*AHA = all-cause hospital admission. CKD = chronic kidney disease. DM = diabetes mellitus. FI = faecal incontinence. IMD = Index of Multiple Deprivation. UI = urinary incontinence. UTI = urinary tract infection.*

## DISCUSSION

### Summary

In this study of 169 524 primary care lower UTI episodes in 86 561 patients using routinely collected data, 3.8% of patients were admitted to hospital for any cause in the following 30 days and 0.8% were admitted for a UTI-related diagnosis. Specific risk factors for UHA included age ≥55 years, recurrent UTI, urinary catheters, CKD, prior antibiotic treatment, FI, and DM. The majority of episodes (77.9%) occurred in female patients, but there was no association between female sex and UHA.

### Strengths and limitations

The strengths of this study include the assessment of microbiological and clinical outcomes to determine the proportion of admissions directly attributable to UTI, rather than simply assessing all-cause admission, which is potentially misleading. The study used a novel database including a large cohort of patients with individual-level data on primary care consultations, antibiotic prescriptions, hospital admissions, and microbiology data, allowing investigation of risk factors for adverse outcomes in a way that has not been done previously. Recognising that coding for clinical consultations is not always complete, a wide range of indicators were used to capture UTI episodes.

The limitations of this study are common to many studies using routinely collected data; the data were collected in short consultations that are focused on delivering clinical care. Prescriptions for UTI-specific antibiotics were used to identify consultations for UTI that did not include a diagnostic Read code. Although nitrofurantoin, pivmecillinam, and fosfomycin are not used for other indications, trimethoprim may rarely be used to treat respiratory tract or skin and soft tissue infections, which may have led to misclassifying consultations for another infection as one for UTI. Positive urine cultures may have represented asymptomatic bacteriuria; however, positive urine cultures accounted for only 5% of the included consultations (Figure 1). Furthermore, UK guidance is to send a urine culture from primary care only in cases where complicated infection is suspected so most urine cultures should be from patients who are symptomatic.^16^

This study aimed to include only community-onset cases and so did not capture attendances or prescriptions from urgent care centres, accident and emergency (without admission), or outpatient clinics. A proportion of included episodes may therefore have been healthcare-associated. Patients with a Read code for a suspected sexually transmitted infection (STI) were not specifically excluded, so it is possible that symptoms were due to STI rather than UTI. However, Read codes included: ‘Urine culture’ (62%), ‘Suspected UTI’ (22%), ‘Urinary tract infection, site not specified’ (8%), and ‘Recurrent UTI’ (3%). Additionally, none of the antibiotics used to identify consultations are used to treat STIs in the UK. In the UK, the majority of STIs are managed in sexual health clinics and a study of conditions treated with antibiotics in primary care in England found that only 6.7% of genitourinary conditions were genital in origin.^15^

The proportion of male patients who were not treated was high, which was surprising given that UTIs in male patients are considered complicated and will usually warrant treatment. It is possible that a significant proportion of the male consultations were catheter urine specimens, which the GP did not treat as they felt a catheter-associated UTI (CAUTI) was clinically unlikely. Very few Read codes for CAUTI were identified, there is no ICD code for CAUTI, and urine cultures frequently do not specify the specimen type. An attempt to mitigate this was made by identifying urinary catheters through prescriptions for devices and accessories recorded in the 6 months preceding the episode, but this will not have captured all CAUTIs. As CAUTI is one of the commonest healthcare-associated infections, this highlights the importance of improving coding of catheter use in primary care. It is also possible that a proportion of men had dysuria that the GP thought was an STI, so a urine culture was sent for completeness and the patient was referred to a sexual health clinic for treatment.

The authors of the study also acknowledge the risk of residual confounding as a study limitation. Non-treatment within +/–7 days was associated with reduced odds of UHA. This is surprising and suggests that the effect of confounding by indication in this study may be stronger than the protective effect of antibiotics in treated patients. There were systematic differences between treated and non-treated patients; non-treated patients were generally younger, had fewer comorbidities, and had less prior antibiotic exposure than treated patients, and this result may simply reflect healthier patients. On examining the data for an interaction between age and antibiotic treatment, no interaction was found (data not shown).

There is evidence that asymptomatic bacteriuria and symptomatic UTI in pregnancy is associated with increased risk of pyelonephritis.^18^ Very few codes related to UTI in pregnancy were identified so it was not possible to examine this as a risk factor.

Furthermore, the population represented in this study is an urban, ethnically diverse, and socioeconomically deprived cohort, and the results may not be generalisable to other settings.

### Comparison with existing literature

This study found that, although patients in the age groups 16–34 years and 35–54 years had the largest proportion of UTI episodes (33.1% and 35.1% respectively; Supplementary Table S1), they did not have greater risk of UHA. This concords with surveillance data on *Escherichia coli* bacteraemia (ECB), where incidence in adults increases with age and is highest in patients aged ≥85 years.^19^^,^^20^ It is also supported by the results of a cohort study using EHRs of all patients at 706 general practices, with 66.2 million person–years of follow up from 2002–2017 and 35 244 first episodes of sepsis, where the risk of sepsis following a consultation for infection was highly age dependent. While the number needed to treat (NNT) to avoid an episode of sepsis was 6517 (95% CI = 4779 to 9522) for men and 13 926 (95% CI = 10 044 to 21 273) for women aged 25–34 years, it was 262 (95% CI = 236 to 293) for men and 385 (95% CI = 352 to 421) for women aged >85 years.^12^

This study found no association between socioeconomic deprivation and UHA, which conflicts with other studies in UTI and other infections.^14^^,^^21^ High levels of deprivation in the study cohort may have affected these results for the rare outcome of UHA. This may also be related to the ability to identify admissions for UTI-specific diagnoses, since the odds of AHA were associated with increasing socioeconomic deprivation.

Trials have examined the safety of withholding antibiotics for UTI in younger women.^10^^,^^11^^,^^22^ Current Public Health England guidance recommends a ‘watch and wait’ approach with a backup antibiotic for likely UTI in non-pregnant women aged <65 years, without a history of recurrent UTI or a urinary catheter.^16^ This study found that CKD, DM, and FI were also associated with increased odds of UHA. A matched cohort study in primary care using Clinical Practice Research Datalink (CPRD) data linked to Hospital Episodes Statistics (HES) data in 242 349 matched pairs of patients found that patients with CKD had higher relative risk of hospital admission with UTI than patients without CKD. On multivariable analysis adjusted for ethnicity, socioeconomic status, smoking status, body mass index, and a number of comorbidities, they found an adjusted hazard ratio of 1.27 (95% CI = 1.23 to 1.32) in patients aged ≤75 years and 1.71 (95% CI = 1.61 to 1.81) in patients aged >75 years.^23^ A number of studies have shown DM to be a risk factor for asymptomatic bacteriuria, symptomatic UTI, pyelonephritis, and ECB.^24^^–^29 Faecal incontinence is associated with increased risk of UTI due to colonisation of the urethra with faecal flora, but the authors have not identified any studies looking specifically at the association with adverse outcomes.

This study found that antibiotic treatment in the previous 6 months was associated with increased odds of UHA. Antibiotic exposure has been associated with increased odds of antibiotic resistance and therefore potential treatment failure, as well as risk of ECB.^24^^,^^30^ A cohort study of 425 women aged 18–40 years at a staff-model health management organisation in the US found the relative risk of developing a UTI was 6.40 (95% CI = 2.43 to 16.84, *P*<0.001) for women who had received antibiotics for UTI in the previous 15–28 days compared with those who had not, and 3.82 (95% CI = 1.95 to 7.45, *P*<0.001) for those who had received antibiotic therapy for a non-UTI indication.^31^

### Implications for research and practice

The study findings suggest that UHA following lower UTI is rare, and there may be scope for reduction in antibiotic prescribing. Studies trialling avoidance or delay of antibiotic treatment may be safely targeted at low-risk groups including patients aged <55 years, without risk factors for complicated UTI, a history of recurrent UTI, or prior antibiotic exposure, and without the comorbidities of DM or FI. Research is needed into the acceptability of such strategies, as avoidance of hospitalisation is likely not to be the only consideration in prescribing decisions.

## Data

Data are not publicly available but are available on request from the researchers.
